# Parenting Styles of Parents Who Had Children With and Without High Risk at Birth: A Cross-sectional Comparative Study

**DOI:** 10.7759/cureus.7079

**Published:** 2020-02-23

**Authors:** Janani Rangarajan, Udayakumar Narasimhan, Abhinayaa Janakiraman, Prajitha Sasidharan, Pavithraa Chandrasekaran

**Affiliations:** 1 Child Development, Sri Ramachandra Institute of Higher Education and Research, Chennai, IND; 2 Developmental Pediatrics, Sri Ramachandra Institute of Higher Education and Research, Chennai, IND; 3 Pediatrics, Sri Ramachandra Institute of Higher Education and Research, Chennai, IND

**Keywords:** authoritarian parent, authoritative parent, father, mother, permissive parent, high risk newborn

## Abstract

Introduction

Parenting style plays a major role in child development by influencing cognitive, social-emotional development, academic performance, and behavioral problems. These characteristics are fairly stable right into adulthood. The influence of risk factors in children on the parenting style of mothers and fathers has not been studied in developing countries.

Aims and methods

The aim of this study is to determine the parenting style of mothers and fathers of children (3-12 years) born with and without high risk and to analyze the influence of this on parenting style. This is an analytical, cross-sectional, comparative study. Sixty-four out of 90 parents of children who have been newborn intensive care unit (NICU) graduates, with moderate to severe risk factors at birth as per the National Neonatology Forum guidelines of India, and 73 parents of children without risk factors at birth were enrolled. A parenting style and dimension questionnaire was used. The commonest parenting style in mothers and fathers and a correlation between parenting style and risk factors in children at birth were identified.

Results

Baseline characteristics were comparable between the high-risk and non-high-risk groups. Eighty percent of mothers and 70% of fathers followed the authoritative parenting style. There was no significant correlation between risk factors and gender, family type or socioeconomic status and the parenting style. Lack of follow-through was the only factor that was significantly present in fathers of children born without risk factors.

Conclusion

Authoritative was the most common parenting style, with no significant difference between parenting in the high-risk and non-high-risk groups. Adopting the appropriate parenting style will optimize developmental outcomes. Further studies are required to look at the influence of proactive positive parenting practices.

## Introduction

The emotional climate in which children are raised by their parents is called parenting style [1]. The two main characteristics of parenting style are the dimensions of parental demandingness (the extent to which parents expect the child to exhibit maturity and responsibility) and responsiveness (the extent of parents being warm and child-centered) [2]. Parenting styles are a combination of these two dimensions [2]. Authoritative, authoritarian, permissive, and indifferent are the four parenting styles derived from these dimensions [2].

Parental direction, disciplinary efforts, and temperament to respond to the child if he or she disobeys are expressed by demandingness while receptive, supportive criticism of the child’s needs is expressed by responsiveness [2]. Parenting style plays a major role in a child by influencing their social-emotional development [3]. Positive parenting practices, such as reading books, should be focused upon in regular pediatric practice, as it improves social-emotional development [4]. Parenting style has an impact on the functioning of the child in terms of social competence, academic performance, psychosocial development, and behavioral problems [5].

The upbringing of the child and psychological factors, such as personality and attachment of parents, are the first two determinants of parenting style. The temperament of the child, which is a feature of the child’s individuality and the stress and support given by the parents is the third determinant of parenting style [6]. Children with risk factors at birth have poorer neurodevelopmental outcomes as compared to children born without high risk. Mothers of children born with high risk factors, especially prematurity, are more prone to develop anxiety and depression, and this can have an impact on parenting style [7-8].

The parenting style and dimensions questionnaire (PSDQ) developed by Robinson et al. in 1995 was used to study the three parenting styles(authoritative, authoritarian, and permissive) [9]. This questionnaire can be used in parents of preschool and school-age children and has been validated in the United States and Turkey [10].

There are only a few Indian studies on parenting style and, currently, no Indian studies are available on the parenting style of children born with high risk factors at birth. There are also no studies discussing the parenting style of fathers in India. Hence, this cross-sectional comparative study was done in mothers and fathers of children born with and without high risk factors at birth. By counseling parents on the appropriate parenting style, we can improve the social, academic, and behavioral outcomes of the child. Differences in developmental outcomes due to a socioeconomic bias can be overcome by strengthening positive parenting practices in the early developmental years [11].

## Materials and methods

This is an analytical, cross-sectional comparative study conducted at Sri Ramachandra Institute of Higher Education and Research after ethical committee approval from August 2018 to May 2019. Ninety parents of children (3-12 years), who were newborn intensive care unit (NICU) graduates with moderate and severe risk factors at birth as per the National Neonatology Forum guidelines of India, attended our child development unit in this time period [12]. They were counseled about the study. Of the 90 parents, 64 parents were enrolled in the study after obtaining consent. Similarly, 73 parents of children from three to 12 years of age, without risk factors at birth, and attending our pediatric outpatient department, were enrolled in the study after obtaining consent. All the parents are literate and conversant in English.

Parents living with the child since birth were included in the study. Parents with chronic medical or psychiatric illness, parents who are separated, and parents of children with a chronic medical illness were excluded from our study. The outcome of the study was to look at the parenting style of both groups of parents of children with and without high risk factors at birth. The demographic information and socioeconomic status (as per the Modified Kuppuswamy Scale) were obtained [13]. Parents were provided with the parenting style and dimensions questionnaire (PSDQ). Authoritative, authoritarian, and permissive are the three parenting styles that can be identified using the questionnaire. The 62 questions in the PSDQ include questions pertaining to these three parenting styles (27 questions based on the authoritative, 20 questions on the authoritarian, and 15 questions on the permissive parenting style), with each question having a rating score from 1 to 5 (1 - never, 2 - once in a while, 3 - half of the time, 4 - very often, 5 - always). Both parents filled the questionnaire individually.

The scoring was done based on the factors under each parenting style. Each parenting style and its factors have a normative mean based on which the parenting style is determined. The calculated mean value is based on the rating of the parents. If the calculated mean value is above the normative mean, it indicates that the parent is following that factor and style. Based on the parenting style and the factors followed under each style, parents were counseled by developmental pediatricians about their current parenting style/s and its consequences. Parents were also educated about the appropriate parenting style as needed. The authoritative parenting style is considered as the best parenting style from previous studies [14]. A mean value above the normative mean for authoritative and below the normative mean for authoritarian and permissive parenting styles is ideal.

Based on previous studies, we estimated the risk difference of parenting styles between both groups as at least 25%. Accordingly, we determined that the number of parents required in both groups was 58 each, based on a power of 80% and an alpha error of 0.05. We performed a statistical analysis using the Statistical Package for the Social Sciences (SPSS) software, version 16 (SPSS Inc., Chicago), and a p-value of less than 0.05 was considered significant. Mean and standard deviation were used for quantitative data analysis and frequency and frequency percentage were used for qualitative data analysis. Chi-square tests were used for association.

## Results

Sixty-four parents of children who were born with high risk (parents of 34 male children and 30 female children) and 73 parents of children who were born without high risk (parents of 35 male children and 38 female children) were included in the study.

Children with and without high risk at birth in our study had no significant difference in terms of age, gender, socioeconomic status, type of family, and order of birth (Table 1). Authoritative was the most common parenting style among both mothers and fathers of children with and without risk factors at birth (Table 2).

**Table 1:** Results of parenting styles and factors of mothers and fathers with or without risk factors at birth

Parenting style	Factors of parenting style	Parents of children born with high risk at birth		Parents of children born without high risk at birth		
		Father	Mother	Father		Mother
Authoritative	Total (Overall Mean-15.026)	42(65.6%)	53(82.8%)	54(74%)		59(80.8%)
	Mean +/- SD	15.81+/-2.64	17.04+/-1.57	16.33+/-2.50		16.64+/-1.85
	P-value					
	Father	0.287				
	Mother	0.763				
	Factor 1-Warmth & Involvement(Mean-4.13)	38(59.4%)	56(87.5%)	43(58.9%)	56(76.7%)	
	Factor 2-Reasoning/Induction(Mean-3.77)	35(54.7%)	48(75.0%)	48(65.8%)	55(75.3%)	
	Factor 3-Democratic Participation(Mean-3.306)	43(67.2%)	55(85.9%)	57(78.1%)	57(78.1%)	
	Factor 4-Good Natured/Easy Going(Mean-3.82)	49(76.6%)	49(76.6%)	59(80.8%)	48(65.8%)	
Authoritarian	Total (Overall Mean-8.62)	36(56.3%)	41(64.1%)	42(57.5%)	47(64.4%)	
	Mean +/- SD	8.90+/-2.79	9.88+/-3.20	9.14+/-2.41	10.36+/-3.27	
	P-value					
	Father	0.88				
	Mother	0.969				
	Factor 1-Verbal Hostility(Mean-2.37)	27(42.2%)	35(54.7%)	36(49.3%)	45(61.6%)	
	Factor 2-Corporal Punishment(Mean-1.8)	35(54.7%)	40(62.5%)	50(68.5%)	49(67.1%)	
	Factor 3-Non Reasoning, Punitive strategies(Mean-1.83)	39(60.9%)	45(70.3%)	51(69.9%)	54(74.0%)	
	Factor 4-Directiveness(Mean-2.62)	29(45.3%)	40(62.5%)	33(45.2%)	39(53.4%)	
Permissive	Total (Overall Mean-6.17)	39(60.9%)	32(50.0%)	39(53.4%)	38(52.1%)	
	Mean +/- SD	6.33+/-1.67	6.17+/-1.72	6.36+/-1.47	6.19+/-1.43	
	P-value					
	Father	0.376				
	Mother	0.81				
	Factor 1-Lack of Follow Through(Mean-2.28)	21(32.8%)	26(40.6%)	43(58.9%)	40(54.8%)	
	Factor 2-Ignoring Misbehaviour (Mean-1.8)	25(39.1%)	24(37.5%)	25(34.2%)	24(32.5%)	
	Factor 3-Self Confidence (Mean-2.09)	42(65.6%)	36(56.3%)	39(53.4%)	44(60.3%)	

**Table 2:** Baseline characteristics

	HIGH RISK	NON-HIGH RISK	P-VALUE
AGE			0.054
3-5 years	36 (56.25%)	33 (45.2%)	
6-9 year	16 (25%)	21 (28.7%)	
10-12 years	12 (18.75%)	19 (26.2%)	
GENDER			0.545
Male	34 (53.1%)	35 (47.9%)	
Female	30 (46.9%)	38 (52.1%)	
SOCIOECONOMIC STATUS			0.076
Class I	11 (17.2%)	22 (30.1%)	
Class II and III	52 (81.3%)	47 (64.4%)	
Class IV and V	1 (1.6%)	4 (5.5%)	
BIRTH ORDER			0.402
1st	48 (75%)	52 (71.2%)	
2nd	12 (18.8%)	19 (26.0%)	
3rd or more	4 (6.3%)	2 (2.7%)	
FAMILY TYPE			0.525
Nuclear	47 (73.4%)	50 (68.5%)	
Joint	17 (26.6%)	23 (31.5%)	

The majority of the parents followed mixed parenting styles; out of which parents following all three styles were the most common. The mixed parenting style was the second most common among mothers of both groups. The mixed parenting style was the second most common in fathers of children born with high risk whereas authoritarian was the second most common in fathers of children born without high risk.

There is no significant difference between the parenting style of parents of children born with and without high risk in our study except father's lack of follow-through, a factor of permissive parenting, which is increased in fathers of children born without high risk (p=0.002).

## Discussion

Parenting style has an important role in child development. Based on the type of parenting, the outcomes may be positive or negative in the personal, social, psychological, and behavioral aspects for children born with or without risk factors. We found that the authoritative parenting style was the most common style in both mothers (80%) and fathers (70%) of children born with and without high risk in our study, similar to studies showing that the authoritative parenting style was predominant in Caucasian, Mexican, and Mexican American families [15-17].

The majority of our parents followed the mixed parenting style (70% of mothers and 60% of fathers), similar to a study done by Marwa et al. [18]. Among the mixed parenting style combinations that were observed, parents following all three parenting styles were the most common. This was followed by the authoritarian parenting style in mothers of children born with high risk and both parents of children without high risk. Studies showed that the authoritarian parenting style was predominant in families with Hong Kong Chinese, Chinese American, and Asian American origin [19].

In our study, the permissive parenting style was the second most common parenting style in fathers of children with high risk. In a study done on Japanese mothers, it was found that they were more lenient and permissive than American mothers [20-21]. We found there was no significant correlation between the gender of the child and the parenting style in both groups. In a study done by Varela et al., Caucasian non-Hispanic mothers and fathers were found to be more authoritarian and Caucasian non- Hispanic fathers were more authoritative when their child was a boy [17].

Analyzing the parenting style of mothers and fathers, authoritative was the most common parenting style and permissive was the least common parenting style. A study conducted by Bamhart et al. on the perception of Indian children on their parent’s parenting style showed that mothers were more authoritative and sometimes permissive, whereas fathers were more authoritarian in contrast to our study [22].

In our study, there is no significant correlation between parenting style and risk factors in children. The parents of children born with risk factors, especially very low birth weight, have more stress, which continues long term, and positive parenting practices help in improving developmental outcomes in children [23-24].

When we look at individual factors within each parenting style, lack of follow-through is the only factor (factor of permissive parenting) that is significantly higher in fathers of children born without high risk than those born with high risk (p=0.002).

There are currently no other Indian studies available that studied the parenting style of fathers.

A positive father-child relationship has been found to be associated with better outcomes and less risk-taking behaviors in children and adolescents. The effects of the authoritarian and permissive parenting styles on children are reduced when fathers maintain a good relationship with the child [25]. Parental warmth, which includes concern or acceptance, supportive presence, positive regard, and emotional support towards the child, has been associated with better behavioral outcomes in high-risk preterm children [26].

In our study, mothers of children born with high risk were good-natured and easy-going, showed more warmth and involvement (87.5%) and democratic participation (85.9%) (factors of the authoritative parenting style) as compared to mothers of children born without high risk but this was not statistically significant. This was similar to a study done by Miles et al. in which mothers gave more attention and time to children born with risk factors mainly prematurity [22].

Parental rejection includes parental anger, hostility, criticism, disapproval, and frustration towards the child. Parental rejection has been associated with poorer behavioral outcomes in children, especially those born with high risk at birth [27]. Factors of the authoritarian parenting style, such as verbal hostility, is comparatively less in mothers of children born with high risk whereas corporal punishment, non-reasoning, and punitive strategies are increased in mothers of both groups. A study conducted by Jambunathan et al. on Asian Indian mothers also reported an increased level of corporal punishment [28].

In our study, directiveness, which is a factor of the authoritarian parenting style is seen at a higher rate in mothers of children with high risk at birth, similar to a study done by Landry et al., which showed that mothers of preterm preschoolers used more directiveness than mothers of children born at term [22].

Self-confidence, which is a factor of the permissive parenting style, is decreased overall in parents of children born with and without high risk in our study. Ignoring misbehavior, which is a factor of the permissive parenting style is more prevalent in mothers of children born with high risk than mothers of children born without risk, similar to a study done by Miles and Holditch-Davis, which showed that mothers of children with risk factors at birth have difficulties in setting limits, are more lenient, and tend to spoil the child [22]. In view of the unique joint family concept in our culture, we looked at whether the parenting style varies in nuclear and joint families but no significant correlation was found between the type of family and parenting style. No other study has looked at this aspect.

We have counseled the parents about the appropriate parenting style and incorporating positive parenting practices, which will have a bearing on future neurodevelopmental outcomes of their children but further follow-up studies will be required to look at the impact of the same. Due to the limitation of the questionnaire usage only from the preschool period, opportunities are lost to counsel parents to follow good parenting practices in early childhood. Since this is predominantly an urban-based study, the parenting style of rural parents also needs to be looked at, although in the digital era, the urban-rural divide may not be a big factor.

## Conclusions

The parenting styles of parents of children with or without high risk factors at birth in our study were not different. The authoritative parenting style is the most common parenting style and the majority of the parents adopt mixed parenting styles. Proper counseling of parents on the appropriate parenting style in early childhood will optimize development in children.
